# Clinical efficacy and safety of beraprost sodium in the treatment of nephrotic syndrome: A meta-analysis

**DOI:** 10.1097/MD.0000000000034958

**Published:** 2023-10-20

**Authors:** Peng Yan, Ben Ke, Xiangdong Fang

**Affiliations:** a Department of Nephrology, The Second Affiliated Hospital of Nanchang University, Nangchang, China.

**Keywords:** beraprost sodium, meta-analysis, nephrotic syndrome (NS), renal function

## Abstract

**Background:**

Beraprost sodium has been shown to have positive effects in the kidney; however, its efficacy and safety in the treatment of nephrotic syndrome (NS) are currently unknown. Therefore, the aim of this meta-analysis was to evaluate the clinical efficacy and safety of beraprost sodium in the treatment of NS.

**Methods:**

We systematically searched EMBASE, PubMed, MEDLINE, China National Knowledge Internet (CNKI), Chinese Biomedical Database (CBM), and Wanfang database for articles from their inception to August 2022.

**Results:**

A total of 12 randomized controlled trials (RCTs) involving 1200 subjects were collected for careful evaluation. The meta-analysis indicated that compared with the controls, combination therapy with berprost sodium could remarkably improve the total effective rate (odds ratio 4.21, 95% confidence interval [CI]: 2.87 to 7.25) and reduce 24 hours proteinuria (mean difference [MD] −1.03, 95% CI: −1.26 to −0.8), serum creatinine (MD −18.39; 95% CI: −27.81 to −8.98), blood urea nitrogen (MD −1.43,95% CI: −1.94 to −0.92), serum total cholesterol (MD −1.24; 95% CI: −1.36 to −1.11), and triglyceride (MD −0.69; 95% CI: −1.03 to −0.35), and increase serum albumin (MD 4.96, 95% CI: 2.98 to 6.93). But the adverse effects of dizziness and headache were higher (RD = 0.05. 95% CI: 0.02 to 0.08).

**Conclusion:**

For NS patients, combination therapy with beraprost sodium can achieve higher clinical efficacy and significant improvement in renal function than conventional therapy.

## 1. Introduction

Nephrotic syndrome (NS) is a clinical disease resulting from increased permeability of the glomerular basement membrane to proteins. The main clinical manifestations of NS are massive proteinuria, hypoproteinemia, hyperlipidemia and peripheral edema, which are recurrent and progressively aggravated. Most cases of NS can be divided into primary and secondary. Minimal change glomerular disease, membranous glomerulonephritis (GN), focal segmental GN, membranoproliferative GN are the main pathological types of primary NS, while secondary causes are mainly associated with drugs and systemic diseases such as diabetes, lupus, hepatitis, and tumors. According to incomplete statistics, the annual incidence of NS in adults is 3 cases per 100,000 people,^[1]^ and although the rate of spontaneous complete or partial remission of NS is above 30%, 10%–30% of patients inevitably progress to end-stage renal disease.^[2]^ This poses a challenge for the treatment and management of NS.

NS has a complex and diverse pathogenesis, usually involving immune mechanisms, inflammation, lipid peroxidation, and other factors. The disruption of the barrier leads to persistent protein leakage, which can lead to interstitial cell injury. Persistent proteinuria promotes kidney injury and is an important factor in the prognosis of chronic kidney disease. Despite the lack of evidence-based guidelines, most patients with NS are suitable for treatment with a low-salt diet, diuresis, reduction of urinary protein, lowering of blood pressure and lipids, and various complications. Immunosuppressive therapy and interventions seem to have promising results. However, long-term steroid and immunosuppressive therapy can bring about serious adverse effects such as nephrotoxicity, hyperglycemia, infections, osteoporosis, etc.

Beraprost sodium (BPS) is a novel and stable synthetic prostaglandin I2 (PGI2) analogue with anti-platelet, anti-proliferative and vasodilatory properties. It has been reported that BPS reduces the vascular resistance of the small glomerular afferent and efferent arteries, regulates renal blood flow, and slows the progression of renal function.^[3]^ BPS can also alleviate renal fibrosis by repairing renal microvasculature and inhibiting the inhibition of inflammatory and oxidative stress effects.^[4]^ In addition, recent studies have demonstrated that Bay also reduces immune complex formation, lowers urinary protein^[5,6]^ and improves the metabolic syndrome in a dose-dependent manner,^[7]^ which is fully consistent with the clinical profile of NS treatment. Several studies have confirmed the renal protective effect of BPS in diabetic nephropathy (DN).^[8,9]^

To date, many clinical practices have reported positive results on the therapeutic effect of BPS for DN, which is highly beneficial for patients. However, the efficacy of BPS in DN has not yet been well appreciated. Therefore, we conducted the first meta-analysis of randomized controlled trials (RCTs) aimed at determining the clinical efficacy and safety of BPS in treating DN.

## 2. Methods

### 2.1. Literature search

Computer search PubMed, Embase, MEDLINE, China National Knowledge Internet (CNKI), Chinese Biomedical Database (CBM) and Wanfang database, and the retrieval language is not limited. Predefined critical search terms included “beraprost” or “beraprost sodium” or “TRK 100” or “TRK-100,” and “Nephrotic syndrome” or “NS.” The search was conducted from the establishment of databases to August 2022, while the references included in the literature were searched manually in order to prevent from neglecting any relevant studies.

### 2.2. Study criteria

The criteria for inclusion in the article were as follows: Study type: studies based on RCTs, no language restrictions. Study population: Patients diagnosed with NS. Intervention: the experimental group was treated with BPS on top of the control group treatment. Outcome indicators: including total effective rate, 24-hour urinary total protein (24-h UTP), serum creatinine (Scr), blood urea nitrogen (BUN),serum albumin (ALB), total cholesterol (TC), triglycerides (TG), and adverse effects. Treatment duration: at least 2 weeks. Exclusion criteria were as follows: review literature, meta-analyses, comments; literature with inaccessible full text and incomplete data or non-convertible data; animal experimental studies; and duplicate publications.

### 2.3. Data extraction

After eliminating the duplicate literature, 2 researchers (Peng Yan, Ben Ke) initially screened the title and abstract according to the inclusion and exclusion criteria, and further read the full text to evaluate the included literature. The general data of the literature were independently extracted according to the designed data extraction table. Data extraction included the first author name, year of publication, age, gender, intervention, control measure, dose, treatment period, outcome. If disagreements arise during the screening and extraction process, agreement is reached through discussion.

### 2.4. Quality assessment

The Review Manager (version 5.3) risk of bias assessment tool was used to assess the study quality of the RCTs. The tool includes 7 entries: participant blinding, personnel blinding, and outcome assessment; random sequence generation; allocation concealment; incomplete data; selective reporting of results; and other biases. Disagreements are resolved through discussion.

### 2.5. Statistical analysis

Review Manager (version 5.3) software and STATA (version 14.0) were used for meta-analysis. Odds ratio (OR) and risk difference (RD) was used for enumeration data, and mean difference (MDs) was used for continuous method data, point estimates and 95% confidence intervals (CI) were given for effect sizes. Q statistic and *I*^2^ test were used to analyze the heterogeneity between studies, which was based on *I*^2^ thresholds of <25%, 25% to 75% and >75% representing mild, moderate and high heterogeneity, respectively. A random-effects model was applied to process when there was heterogeneous results, whereas a fixed-effects model was used for poor heterogeneity. *P <* .05 was considered statistically significant in this meta-analysis. We analyzed the data using subgroup and sensitivity analyses to explore potential sources of heterogeneity. Publication bias for these studies was evaluated using Egger and Begg test funnel plots.

### 2.6. Ethics approval

This study is a systematic review and the content does not involve ethical approval or unethical items.

## 3. Results

### 3.1. Description of included studies

**Figure 1.**
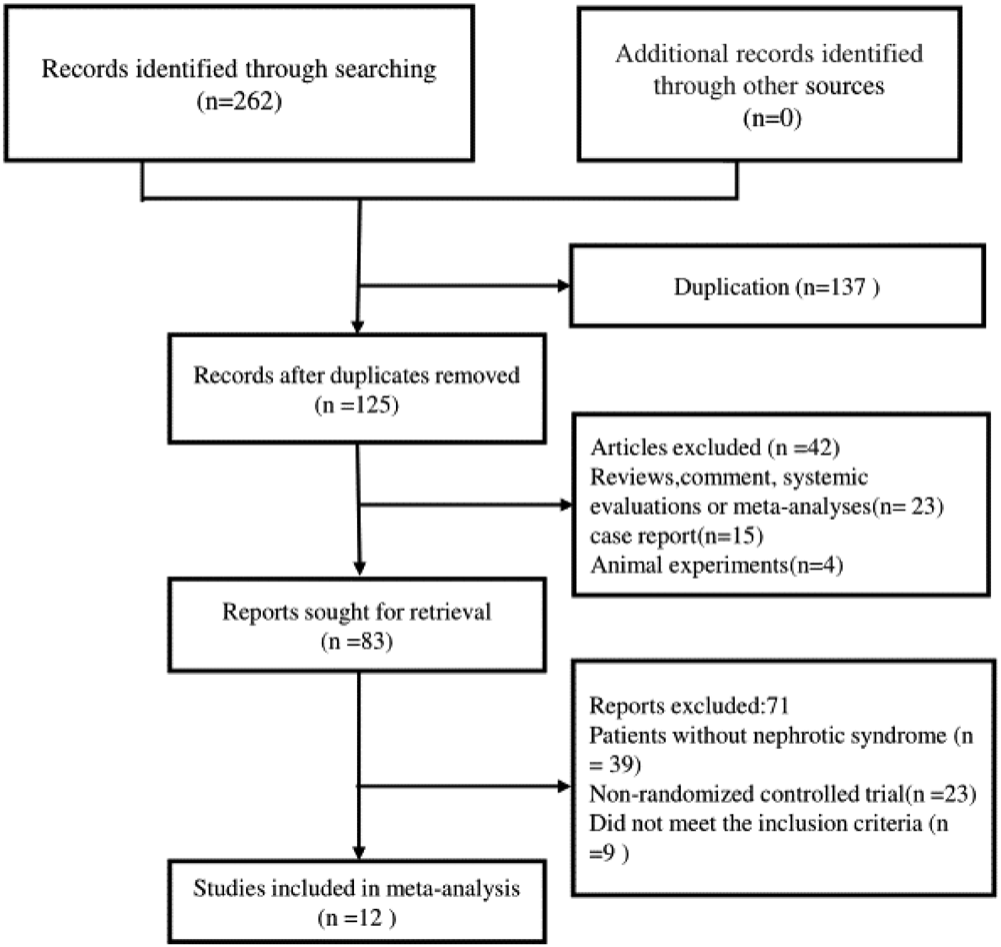
Study selection flow diagram.

A preliminary search of the database yielded 262 Chinese and English articles. After removing duplicates, reading titles and abstracts, and detailing the full text, 12 studies^[10–21]^ with a total of 1200 patients were finalized. Six hundred of them belonged to control group while 600 of them were in the test group (Fig. 1 shows the literature screening process). All studies were performed and published in China between 2018 and 2022.The number of participants in each study ranged from 50–127, and the duration of treatment varied from 2 weeks to 12 months. The control group measures consisted of conventional and immunosuppressive treatment, while the experimental group was intervened with BPS on top of the control group. All of the doses used for Beraprost were 40 micrograms 3 times daily, except one which was 40 micrograms once a day.^[12]^ More details are shown in Table 1.

**Table 1:** The characteristics of included studies.

Study year	Age (yr)		Gender (male/female)	Course	Control group method	Test group method	BPS Dose	Treatment period	Outcome
	T	C							
Huang, 2009	40.25 ± 3.63		26/24	8.32 ± 2.43 mo	Methylprednisolone + tacrolimus	+ BPS	40 µg 3 times/d	6 mo	①②③④
Li, 2021	50.34 ± 11.07	48.69 ± 11.85	67/47	2–16 mo	Tacrolimus	+ BPS	40 µg 3 times/d	6 mo	①③⑤⑥⑦
Lin, 2019	51.2 ± 12.2	51.3 ± 12.8	71/49	3–14 mo	Tacrolimus + aspirin	+ BPS	40 µg 3 times/d	6 mo	①②③⑤
Liu, 2022	43.68 ± 6.30	43.93 ± 6.22	69/55	7.64 ± 1.20 mo	Hormone + tacrolimus	+ BPS	40 µg 1 times/d	2 wk	①②③④⑤
Peng, 2021	44.35 ± 6.97	43.66 ± 6.25	79/39	1–8 yr	Prednisone	+ BPS	40 µg 3 times/d	6 mo	①③④⑤
Wang, 2018	49.32 ± 3.80	45.05 ± 4.02	60/26	3.82 ± 0.86 yr	Basic treatment + immunosuppressor	+ BPS	40 µg 3 times/d	12 mo	①②③⑤⑥⑦
Wang, 2019	49.9 ± 9.2	49.8 ± 9.3	49/31	2–10 mo	Prednisone + tacrolimus; aspirin	+ BPS	40 µg 3 times/d	6 mo	①②③④⑤⑥⑦
Wang, 2020	43.58 ± 6.24	44.24 ± 7.02	78/38	4.05 ± 1.33 yr	Prednisone	+ BPS	40 µg 3 times/d	6 mo	①②③④⑤⑥⑦
Wang, 2021	60.12 ± 2.84	60.21 ± 2.78	46/44	14.5 ± 3.1 mo	Methylprednisolone	+ BPS	40 µg 3 times/d	6 mo	②③④⑥⑦
Xiao, 2022	63.85 ± 14.10	63.85 ± 14.10	NA	2.61 ± 0.72 yr	Prednisone	+ BPS	40 µg 3 times/d	6 mo	①②③④⑤
Zhao, 2022	40.41 ± 10.03	42.03 ± 8.62	51/44	1–10 mo	Prednisone + methylprednisolone	+ BPS	40 µg 3 times/d	3 mo	①③④⑥⑦
Zhu, 2022	66 ± 4	66 ± 4	75/52	5–15 mo	Tacrolimus	+ BPS	40 µg 3 times/d	2 mo	②③④⑥⑦

Outcomes: ① total effective rate, ② 24-h urinary protein, albumin, ③ Serum creatinine (Scr), ④ blood urea nitrogen (BUN), ⑤ serum albumin (ALB), ⑥ total cholesterol (TC), and ⑦ triglycerides (TG).

BPS = beraprost sodium, C = control group, T = treatment group.

### 3.2. Methodological quality

**Figure 2.**
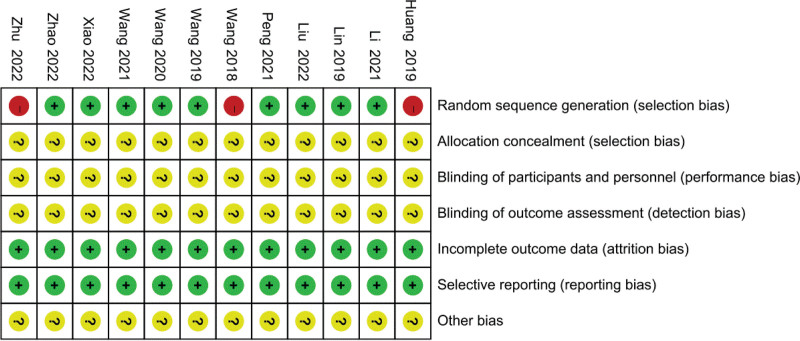
Quality assessment summary.

All included studies mentioned randomization, 9 of which described specific randomization methods and were rated as low-risk studies, while 3 studies were rated as high-risk studies due to uncritical randomization methods. None of the literature addresses specific assignment concealment or blinding. Figure 2 reveals the results of the risk of bias assessment.

### 3.3. Primary outcome measures

#### 3.3.1. Meta-analysis of the total effective rate.

**Figure 3.**
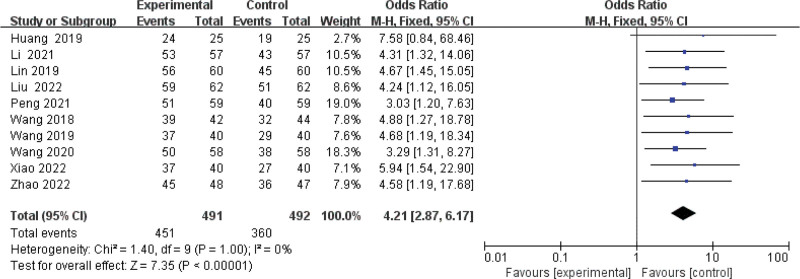
Forest plot of meta-analysis of the total effective rate of beraprost sodium in NS treatment. NS = nephrotic syndrome.

Ten publications^[10–16,18–20]^ investigated the effect of total effective rate after treatment. As shown in Figure 3, results from this meta-analysis showed that the pooled OR was 4.21 (95% CI: 2.87 to 6.17; *P* < .00001) among the 10 articles, which suggested that using BPS as a combination treatment was more effective than control group treatment in treating NS. Due to a low heterogeneity (*P* = 1.0, *I*^2^ = 0%), the fixed-effect model was used for this meta-analysis.

#### 3.3.2. Meta-analysis of the 24-hour UTP level.

**Figure 4.**
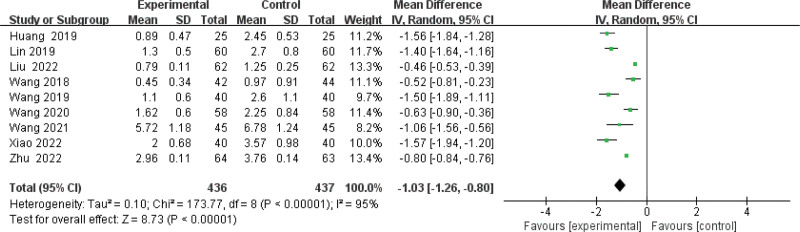
Forest plot of meta-analysis of 24-h urinary protein difference of beraprost sodium in NS treatment. NS = nephrotic syndrome.

Nine publications^[10,12,14–19,21]^ evaluated 24-hour UTP in the combination and control groups. After a test of heterogeneity (*P* < .00001, *I*^2^ = 92%), we used a random-effects model. As shown in Figure 4, the results showed a statistically significant comparison of the efficacy of the combination group in significantly reducing 24-hour proteinuria compared to the control group (MD −1.20, 95% CI: −1.40 to −0.83, *P* < .00001). We performed a sensitivity analysis for 24-hour urine protein and the results of the sensitivity analysis were stable (Supplementary Figure 1, http://links.lww.com/MD/J661). Results of Egger test (*P* = .218) suggested that there were no significant publication biases among the 9 reports(Supplementary Figure 2, http://links.lww.com/MD/J662).

To explore the source of heterogeneity, we performed a subgroup analysis based on clinical characteristics. The results showed that the effect of combination therapy with BPS on the 24-h UTP level was consistent with the results in each subgroup, whether it was the treatment regimen, duration of treatment, or duration of the patient disease (Table 2). In addition, in studies with subgroups divided into at least 6 months (MD −0.63, 95% CI: −0.96 to −0.30, *I*^2^ = 98%) and <6 months(MD −1.17, 95% CI: −1.53 to −0.82, *I*^2^ = 89%), the results revealed that it was more significant in reducing urinary protein for the combined group compared with the control group when the course of treatment was t ≥ 6 months (*P* = .03). Unfortunately, we were unable to remove heterogeneity from the subgroup analysis.

**Table 2:** Sensitivity and subgroups analysis based on 24-h urinary protein.

	Group	No. of reports	No. of patients	MD	95% CI	Z	*P* (effect)	*I* ^2^	*P* (overall)
Treatment course	<6 mo	2	251	−0.63	−0.96, −0.30	3.71	= .0002	98%	.03
	≥6 mo	7	622	−1.17	−1.53, −0.82	6.47	<.00001	89%	
Disease course	<2 yr	6	591	−1.10	−1.38, −0.82	7.65	<.00001	96%	.54
Baseline urine protein(24h)	≥2 yr ≥8 g/L <8 g/L	3 4 5	282 336 537	−0.89 −1.24 −0.84	−1.48, −0.31 −1.72, −0.76 −1.10, −0.59	3.01 5.08 6.49	<.0001 <.00001 <.00001	90% 91% 96%	.15
Treatment modes	GC + TAC/CYC	3	254	−1.16	−2.02, −0.30	2.65	<.00001	97%	.12
	GC alone	2	196	−1.09	−2.01, −0.17	2.32	<.00001	94%	
	TAC/CYC	3	337	−1.08	−1.53, −0.62	4.64	<.00001	92%	
	No limitation	1	86	−0.52	−0.81, −0.23	3.54	-	-	

MDs = mean difference.

#### 3.3.3. Meta-analysis of Scr level.

**Figure 5.**
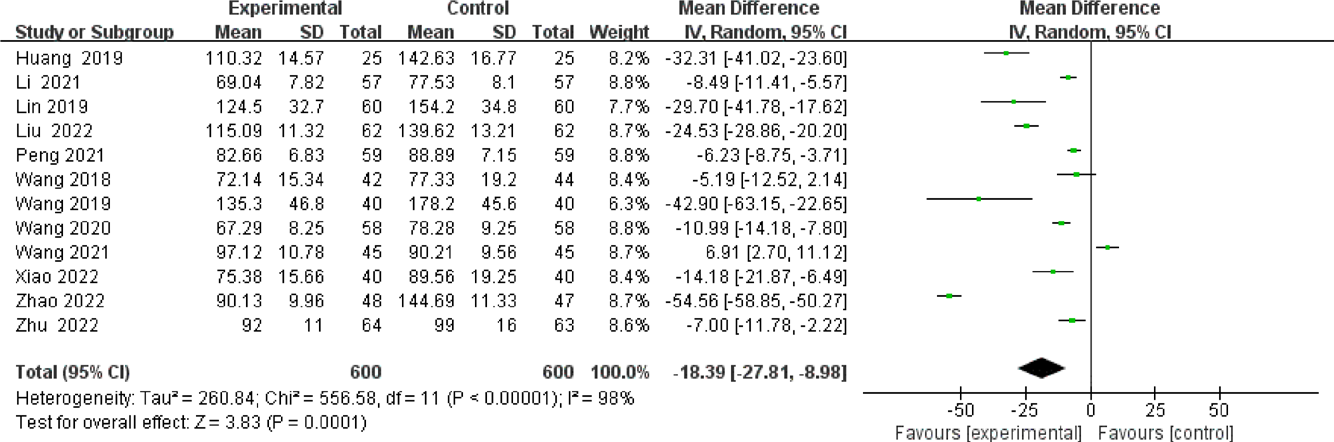
Forest plot of meta-analysis of serum creatinine difference of beraprost sodium in NS treatment. NS = nephrotic syndrome.

**Figure 6.**
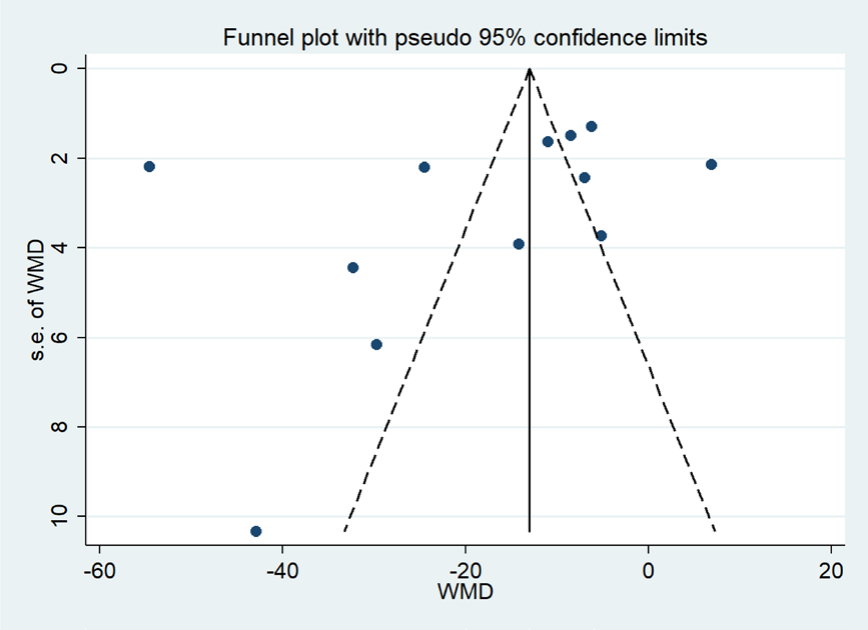
Funnel plot of serum creatinine.

Twelve publications^[10–21]^ have determined the impact of BPS supplementation in the treatment of patients with NS. After testing for heterogeneity (*P* < .00001, *I*^2^ = 98%), a random effects model was chosen for data analysis. As shown in Figure 5, results indicated that the use of BPS in combination therapy significantly reduced Scr (MD −18.39, 95% CI: −27.81 to −8.98,*P* = .0001) than the control. Potential publication bias in 12 studies was evaluated by funnel plots and the results indicated a slight asymmetry as shown in Figure 6. We used Egger test to assess publication bias and a *P (P* = .340) > 0.05 was considered to have no publication bias (Supplementary Figure 3, http://links.lww.com/MD/J663). To assess whether the overall efficacy of BPS in the treatment of Scr is affected by low-quality RCTs, a sensitivity analyses we conducted showed that the omission of each individual study did not have a significant effect on pooled effect size (Supplementary Figure 4, http://links.lww.com/MD/J664).

Due to the high heterogeneity, different duration of treatment and disease were used for subgroup analysis in our study. As shown in Supplementary Figure 5, http://links.lww.com/MD/J665, the treatment duration subgroup showed that the combined group was superior to the control group in reducing Scr (“at least 6 months”: MD = −13.08, 95% CI: −19.14 to −7.02, *I*^2^ = 74%; “less than 6 months”: MD = −28.71, 95% CI: −55.78 to −1.65, *I*^2^ = 99%).The effect of subgroup of disease duration < 2 years was used (MD = −17.03, 95% CI: − 18.72 to − 15.33, *I*^2^ = 99%) is more significant than at least 2 years (MD = −8.23, 95% CI: − 10.09 to − 6.38, *I*^2^ = 64%), the results were statistically significant (*P* < .00001) (Supplementary Figure 6, http://links.lww.com/MD/J666). However, significant heterogeneity remained within each subgroup.

#### 3.3.4. Meta-analysis of BUN level.

**Figure 7.**
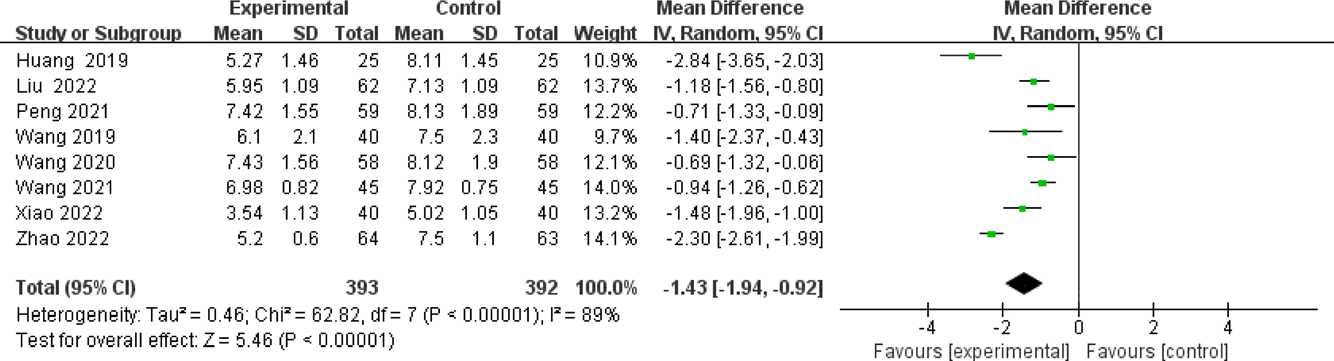
Forest plot of meta-analysis of blood urea nitrogen difference of beraprost sodium in NS treatment. NS = nephrotic syndrome.

Eight articles^[10,13–17,19,21]^ suggested a difference in BUN between the combined and control groups. Owing to the clear heterogeneity among studies (*I*^2^ = 89%), therefore, a random effects model was used for estimate the MD. The level of BUN was significantly reduced in the combined group compared with the control group (MD −1.43, 95% CI: −1.94, −0.92, *P <* .00001) (Fig. 7), and the subgroup according to treatment course <6 months (MD = −13.08, 95% CI: −19.14 to −7.02, *I*^2^ = 98%) or at least 6 months (MD = −13.08, 95% CI: −19.14 to −7.02, *I*^2^ = 98%) also match the same result (Supplementary Figure 7, http://links.lww.com/MD/J667). The results of sensitivity analysis were robust (Supplementary Figure 8, http://links.lww.com/MD/J668).Egger test (*P* = .763) showed that there was no publication bias (Supplementary Figure 9, http://links.lww.com/MD/J669).

#### 3.3.5. Meta-analysis of serum albumin levels.

**Figure 8.**
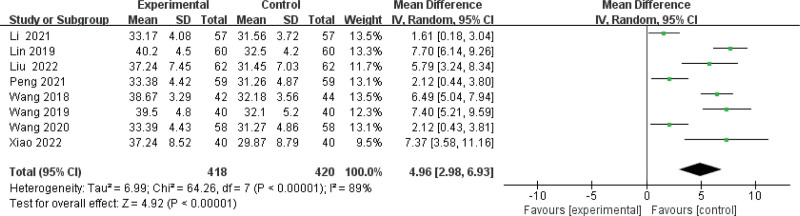
Forest plot of meta-analysis of serum albumin levels difference of beraprost sodium in NS treatment. NS = nephrotic syndrome.

Eight studies^[11–16,18,19]^ reported changes in serum albumin levels in the combined and control group. A random effects model (*I*^2^ = 89%) was used to estimate MD, and the overall MD was 4.96 (95% CI: 2.98 to 6.93, *P* < .00001) Figure 8, which shows a significant difference between BPS supplementation and control group. The result was also supported by the subgroup analysis based on treatment course <6 months (MD 5.79; 95% CI: 3.24 to 8.34; *I*^2^ = 89%) or at least 6 months (MD 4.86; 95% CI: 2.68 to 7.04; *I*^2^ = 91%) (Supplementary Figure 10, http://links.lww.com/MD/J670). There was an evidence of publication bias (Egger test: *P* = .496) and the effect size was robust in sensitivity analysis(Supplementary Figure 11, http://links.lww.com/MD/J671,12, http://links.lww.com/MD/J672).

#### 3.3.6. Meta-analysis of blood lipid levels.

**Figure 9.**
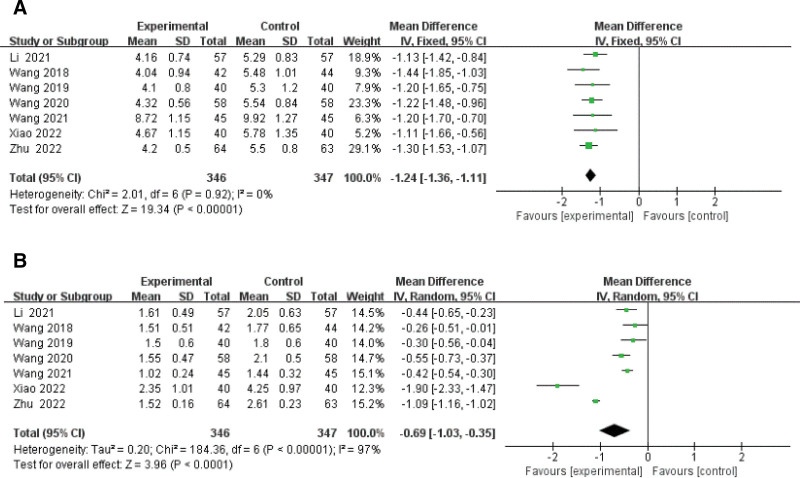
Forest plot of meta-analysis of lipid levels difference of beraprost sodium in NS treatment. (A) Total cholesterol and (B) triglycerides. NS = nephrotic syndrome.

A forest plot of the results of the meta-analysis of blood lipid summaries is shown in Figure 9. We observe a significant effect of BPS intake on lipid levels, including total cholesterol (MD −1.24, 95% CI: −1.36 to −1.11, *I*^2^ = 0%) and triglycerides (MD −0.69, 95% CI: −1.03 to −0.35, *I*^2^ = 97%) in 7 studies.^[11,15–19,21]^

### 3.4. Adverse reaction

Eight^[11–16,18,20]^ of the trials described adverse events in detail, while the others did not mention adverse events. Eight studies noted gastrointestinal discomfort, 5 studies mentioned infection, 4 studies referred to liver damage, 3 papers mentioned elevated blood glucose, and 2 mentioned embolism, all of which were present in both experimental and control groups and were not statistically significant(Table 3). The incidence of dizziness and headache was higher in the combined BPS group than in the control group (RD = 0.05. 95% CI: 0.02 to 0.08, *P* = .002) (Supplementary Figure 13, http://links.lww.com/MD/J673), these symptoms are usually self-limiting and do not affect treatment. Hence, BPS was safe in treating NS.

**Table 3:** Incidence rate of adverse reaction.

Type	Number of adverse reactions		References
	Treatment group control group		
Headache and dizziness	26	8	^[11–16,18]^
Gastrointestinal discomfort	23	32	^[11–16,18,20]^
Infection	24	32	^[13,15,16,18,20]^
Hepatic lesion	9	6	^[13–15,18]^
Elevating blood glucose	9	8	^[13,15,18]^
Thromboembolism	2	4	^[15,18]^

## 4. Discussion

In NS, impairment of the charge barrier and size barrier results in leakage of proteins into the urine. Persistent proteinuria is one of the most important adverse prognostic factors in the progression of chronic kidney disease to end-stage renal failure.^[22,23]^As a result, aggressive reduction or elimination of proteinuria has been recognized as a goal in the treatment of NS. Immunosuppressive therapy is the main treatment used for NS patients. The most commonly used hormones can inhibit the immune inflammatory response, inhibit the secretion of aldosterone and antidiuretic hormone, affect the permeability of the glomerular basement membrane, and play a role in eliminating proteinuria. However, long-term use of immunosuppressive agents not only brings obvious side effects, but also leads to ineffective immunodrug therapy due to etiological heterogeneity, and some patients inevitably relapse after treatment.

BPS is an orally active agent of prostacyclin(prostaglandin I2/PGI2) analog, like most PGI2, BPS has antiplatelet and vasodilatory effects, mainly due to the inhibition of Ca^2+^ influx and thromboxane A2 production by increasing intracellular concentrations of cyclic adenosine monophosphates. Therefore, in Asian countries, it has been successfully used to improve intermittent claudication and pain caused by chronic arterial occlusive disease and to treat primary pulmonary hypertension.

Recently, increasing evidence has shown that BPS has distinct advantages in the treatment of kidney disease, whether it is to protect against acute kidney injury such as contrast medium nephropathy and cisplatin nephropathy^[24,25]^ or to delay the progression of chronic kidney disease (CKD) by maintaining renal microvascular blood flow.^[4,26]^ In addition, some pharmacological and clinical trials on the efficacy and mechanism of BPS in treating nephropathy have demonstrated the important role of BPS in reducing proteinuria, improving metabolism and protecting renal function. Improving renal perfusion. BPS can reduce the vascular resistance of glomerular afferent and efferent arteries regulating the renal hemodynamics without glomerular hyperfiltration.^[3]^ Inhibiting mesangial cell growth. BPS alleviates excessive constricting effect of angiotensin II on renal vasculature, inhibits mesangial growth via platelet-derived growth factors, and reduces leakage of urinary.^[27]^ Reducing the permeability of the renal vasculature. BPS inhibits inflammatory cytokines produced by monocytes/macrophages,^[6]^ reduces immune complex deposition in glomeruli and protects endothelial cells,^[5]^ thereby improving vascular permeability and diminishing urinary protein. Anti-renal fibrosis. BPS reduces inflammation and oxidative stress in kidney tissue, suppressing endothelial-mesenchymal transition and collagen gene expression, thereby alleviating renal fibrosis and benefiting kidney recovery.^[28]^ Metabolic management. BPS plays a role in regulating metabolism by improving insulin resistance and glucose intolerance and suppressing triglyceride and cholesterol levels.^[7,29]^ BPS has a positive effect on reducing proteinuria nephropathy, but its efficacy in the treatment of NS needs to be further reviewed and analyzed.

In the present meta-analysis, we found that patients who received BPS combined standard therapy were more effective in reducing proteinuria, increasing total response rate and serum albumin, improving Scr, BUN, and blood lipids than those who only received standard therapies. The risk of adverse effects was not significantly elevated, except for dizziness and headache which were 5% higher in the combination group than in the control group, and these were minor and did not affect the trial. In order to explore the heterogeneity of the meta-analysis, we performed subgroup analyses according to the different treatment procedures. We observed a greater benefit of prolonging the combination course of BPS (≥6 months) in reducing the 24-hour urinary protein subgroup compared to the control. However, improvements in SCr, BUN, lipids and serum albumin in subgroups with disease duration did not appear to be statistically significant. Considering the low quality of these studies and the relatively small number of cases included, the reliability of this finding needs to be further validated by prospective studies. In the Scr treatment subgroup analysis, the combination of BPS showed a more significant effect on patients with a disease duration of <2 years compared to the control. Given the lack of specificity for immunosuppressive drugs and the ambiguity of the detailed drug regimens in the included studies, their reliability needs further confirmation and this finding should be carefully interpreted. We also performed sensitivity analyses for total effective rate, 24-h UTP, Scr, BUN, and serum albumin levels, and the results showed that our meta-analysis had low sensitivity and high stability, in addition to Begg and Egger test funnel plots showing no publication bias.

Except for low heterogeneity in total effective rate and total cholesterol, the significant heterogeneity in other metrics of the included the studies in the meta-analysis was existed and unavoidable, although we performed subgroup and sensitivity analyses to explore possible sources of heterogeneity but failed, which may be attributed to the following reasons: Different treatments have been used in different studies, with some studies using hormones alone in the control group, some tacrolimus, some cyclophosphamide, and even some studies including all of them. In addition, the means of conventional treatment were inconsistent. Different studies had different inclusion criteria; for example, some studies selected primary NS and some did not describe it. Patients had different characteristics in different regions, for example, some studies had NS for only a few months and some for 4 years. All of these may account for the high degree of heterogeneity, although subgroup analyses of treatment duration and patient disease duration were performed, however, heterogeneity was not significantly reduced.

There are several limitations to this meta-analysis. First of all, all of the studies were small samples, and studies with larger sample sizes are needed. Second, all included studies were collected in Chinese journals, which may have some language bias as well as limit our generalization of the findings. However, the positive effects of sodium berprost in NS should not be underestimated worldwide. Third, we lack knowledge of the pathology of NS under these studies, which has weak statistical power for treatment outcomes of different pathological types. This factor may be one of the causes of heterogeneity. Fourth, the current study has a short duration of treatment; therefore, the long-term effects of BPS remain debatable. Finally, the quality of included RCTs was generally poor, such as some randomization methods were not standardized, hidden allocation and blinding unknown, and inadequate study design.

## 5. Conclusion

In conclusion, the results of this meta-analysis suggest that the combination of BPS is more effective in treating NS than using conventional immunosuppressive therapy alone, which has a promising application. However, the included studies had a small sample size and significant statistical heterogeneity, so more stringent large-sample multicenter RCTs are needed for further exploration and confirmation.

## Author contributions

**Conceptualization:** Peng Yan.

**Data curation:** Peng Yan.

**Formal analysis:** Peng Yan.

**Investigation:** Ben Ke.

**Methodology:** Ben Ke.

**Project administration:** Ben Ke.

**Supervision:** Dong Xiang Fang.

**Validation:** Dong Xiang Fang.

**Visualization:** Dong Xiang Fang.

## Supplementary Material


























