# Supplementary figures and images for: Clinical efficacy and safety of beraprost sodium in the treatment of nephrotic syndrome: A meta-analysis
Source: Medicine (Baltimore). 2023 Oct 20;102(42):e34958. doi: 10.1097/MD.0000000000034958 (PMC10589524; doi:10.1097/MD.0000000000034958)

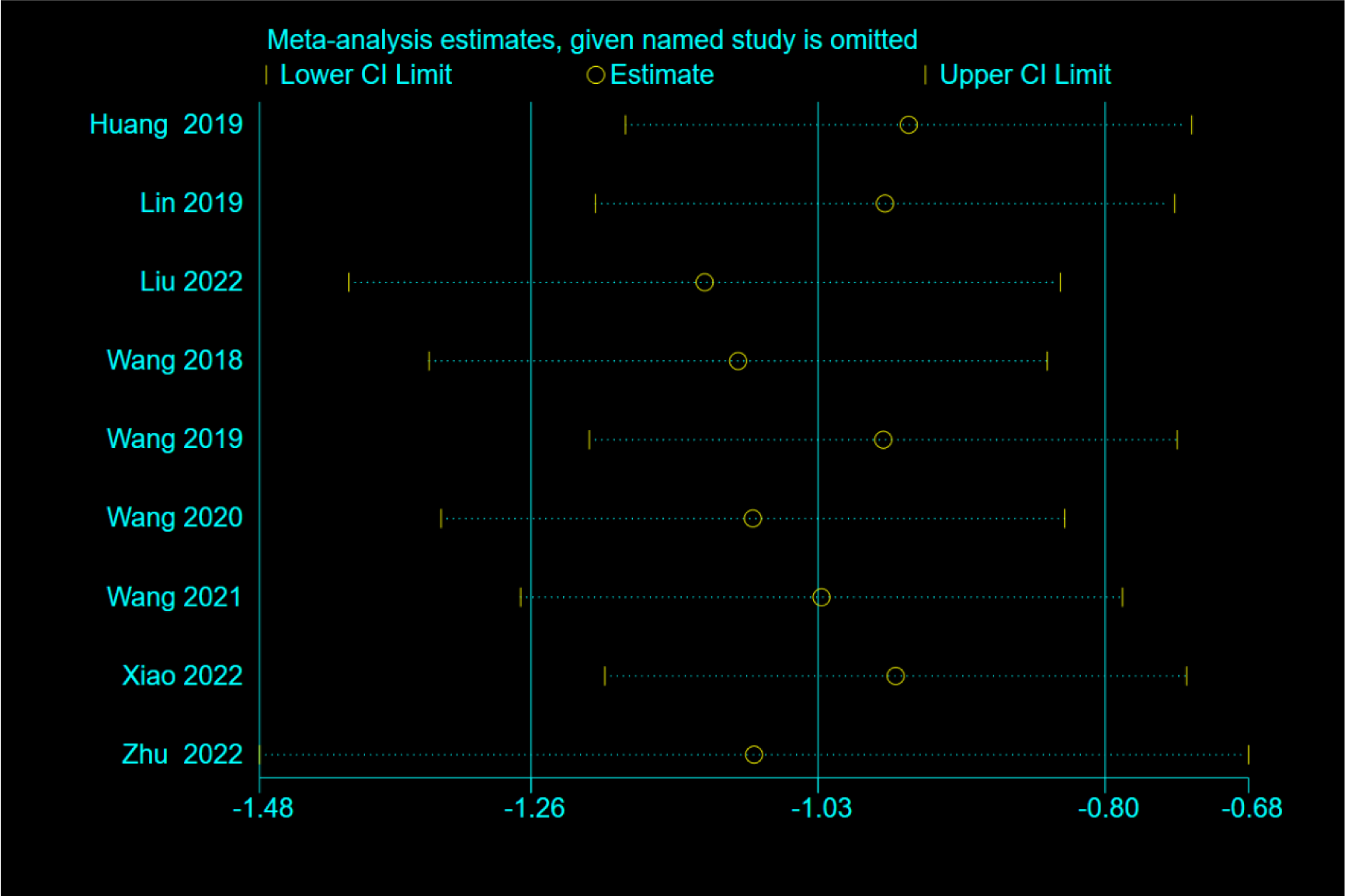

Supplement: Supplementary file 1 [file medi-102-e34958-s001.tif]

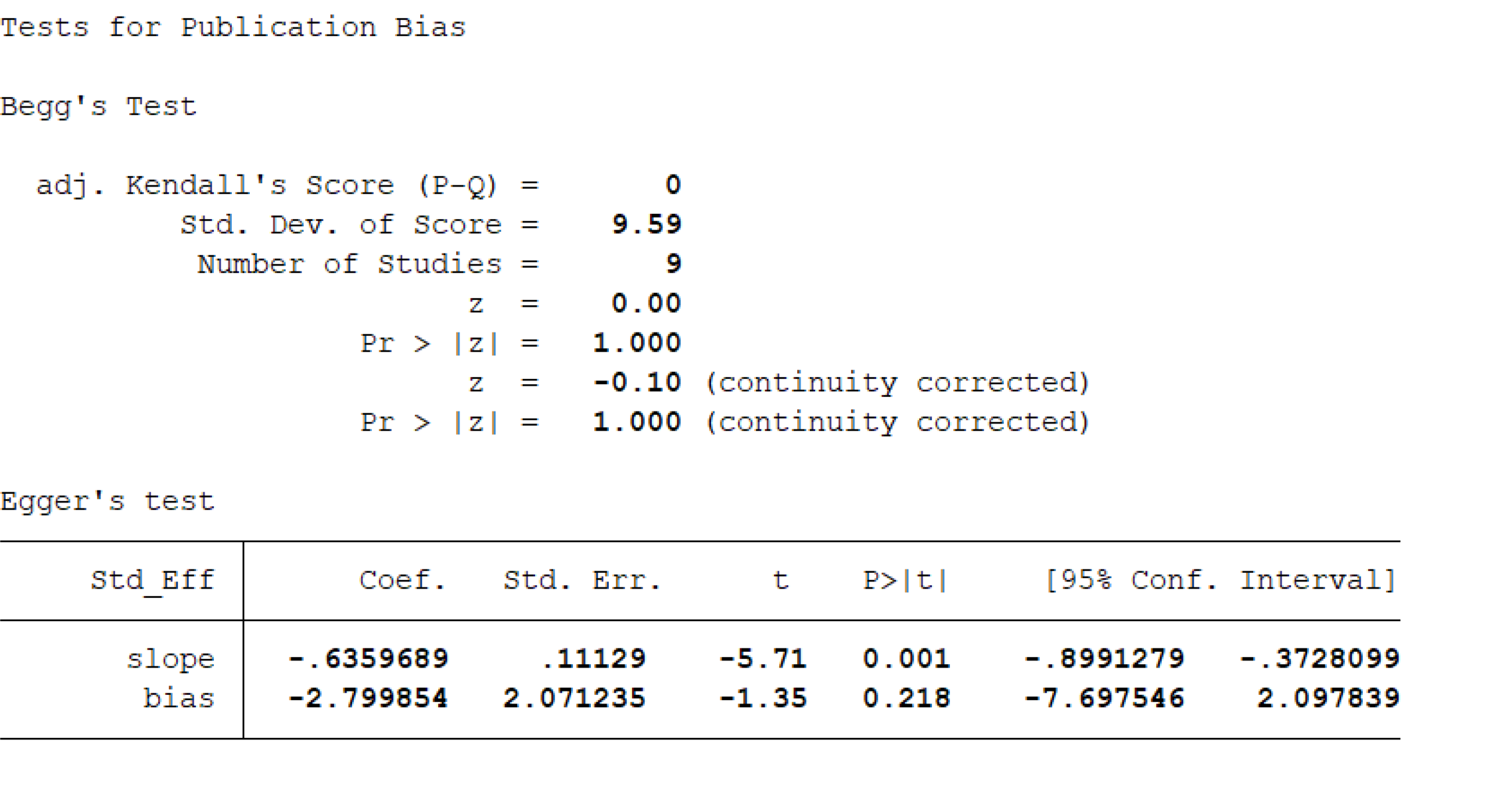

Supplement: Supplementary file 2 [file medi-102-e34958-s002.tif]

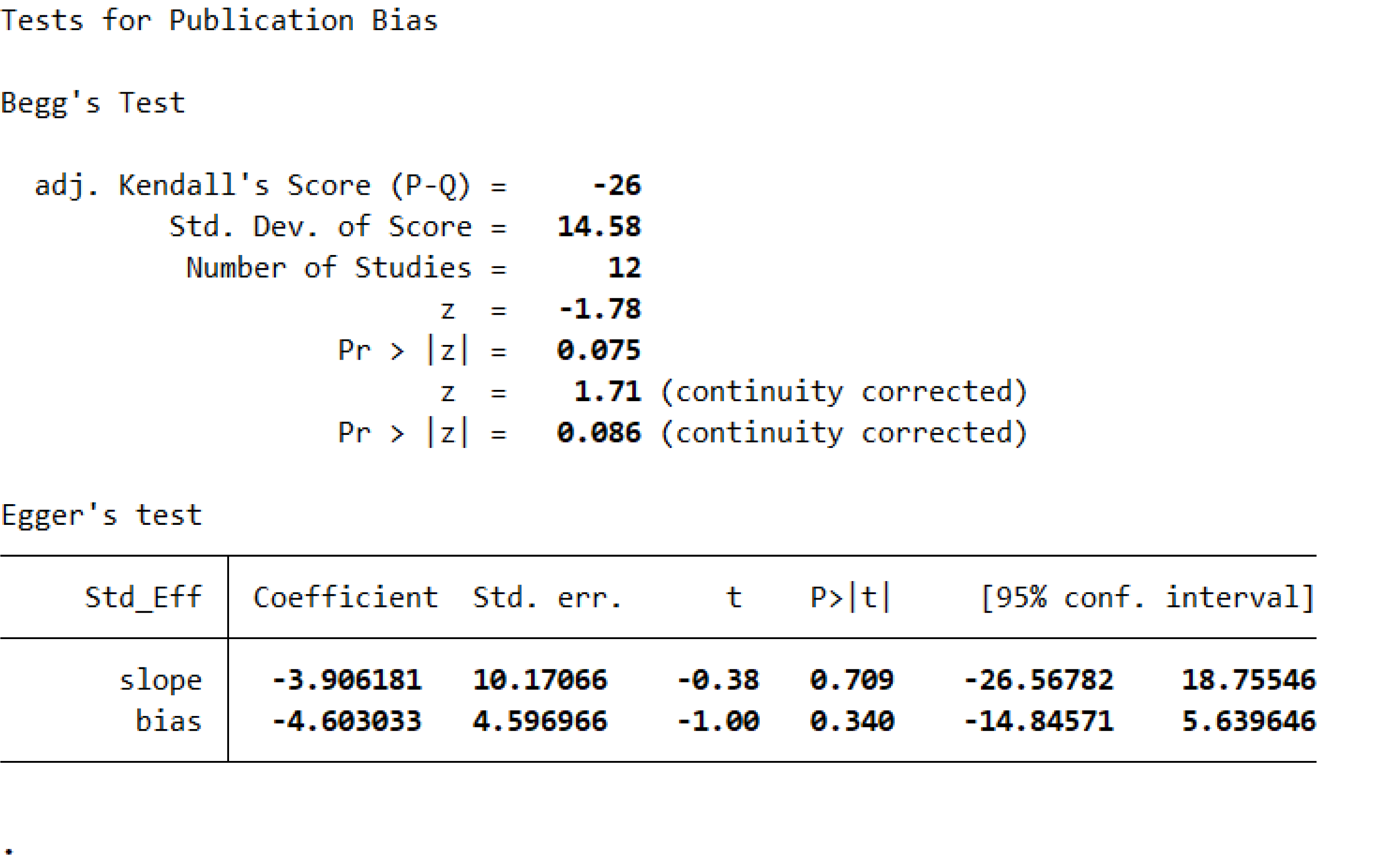

Supplement: Supplementary file 3 [file medi-102-e34958-s003.tif]

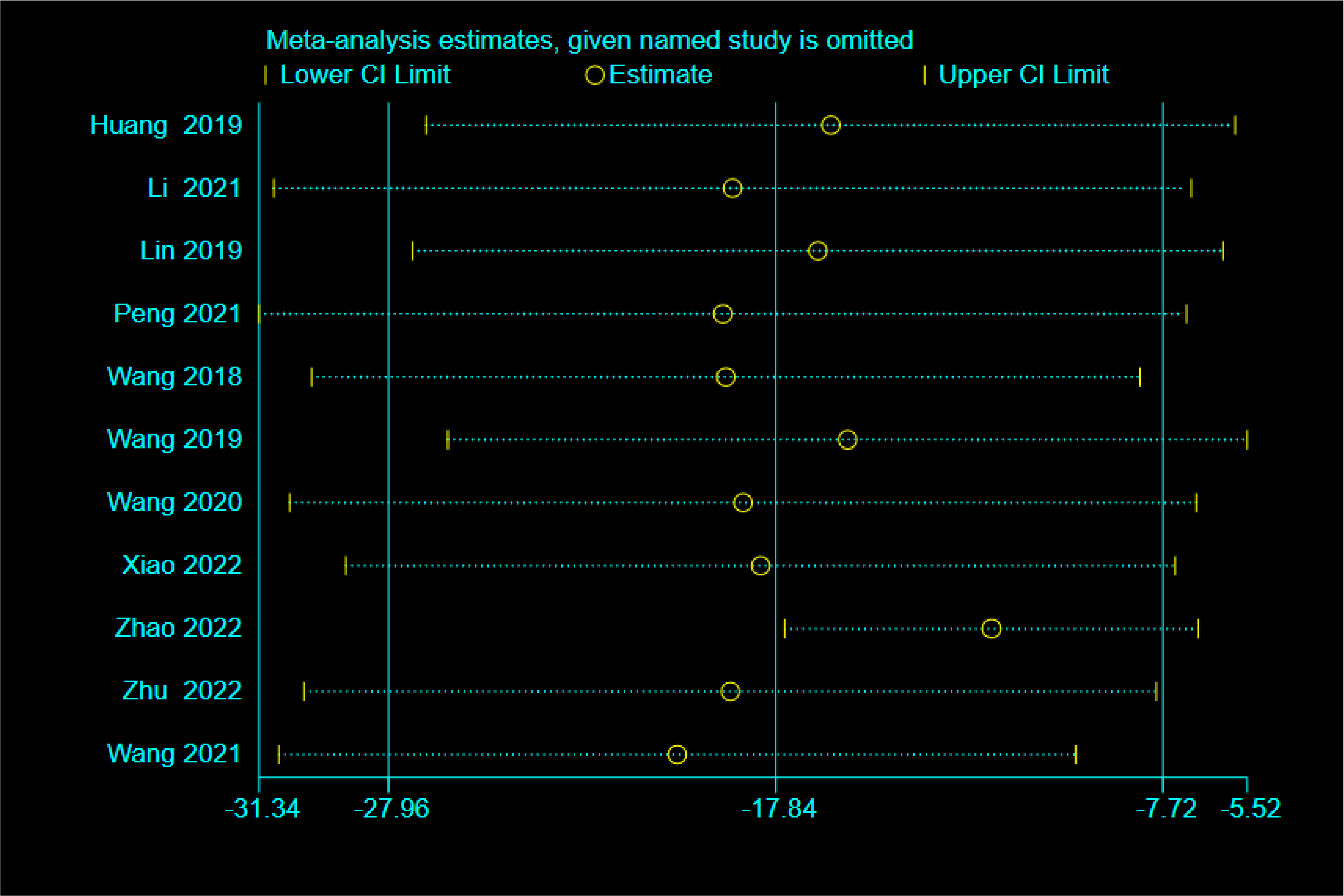

Supplement: Supplementary file 4 [file medi-102-e34958-s004.tif]

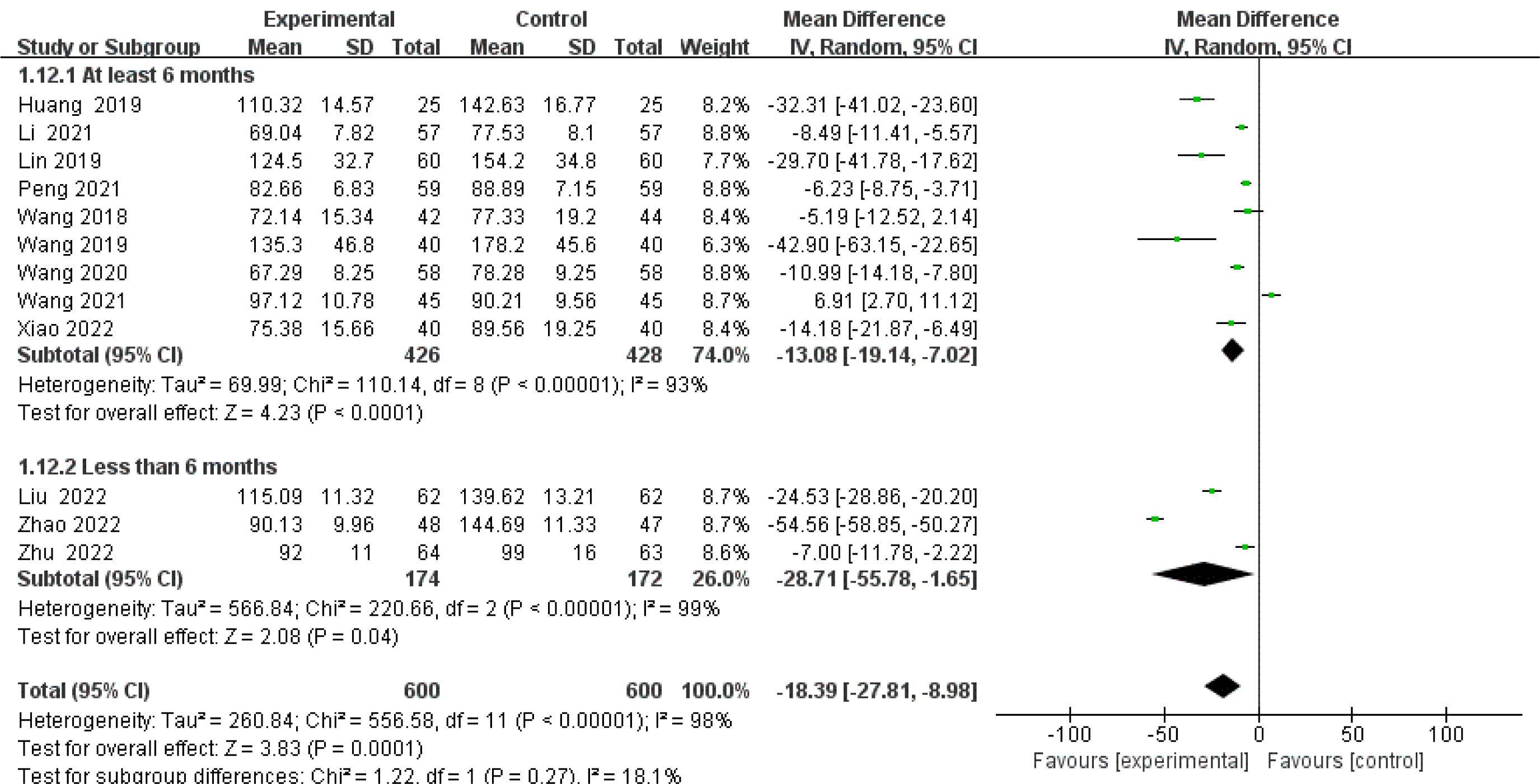

Supplement: Supplementary file 5 [file medi-102-e34958-s005.tif]

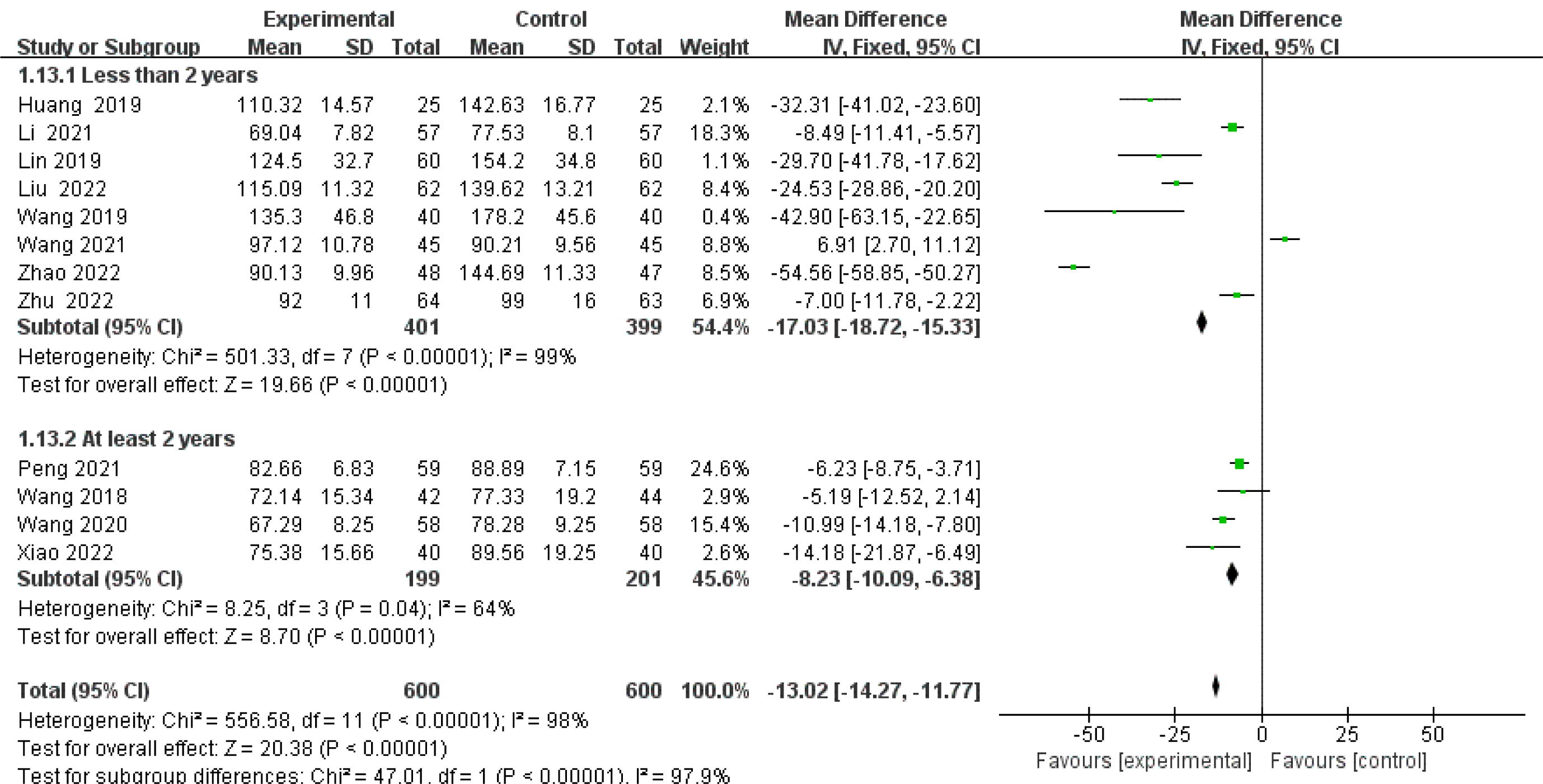

Supplement: Supplementary file 6 [file medi-102-e34958-s006.tif]

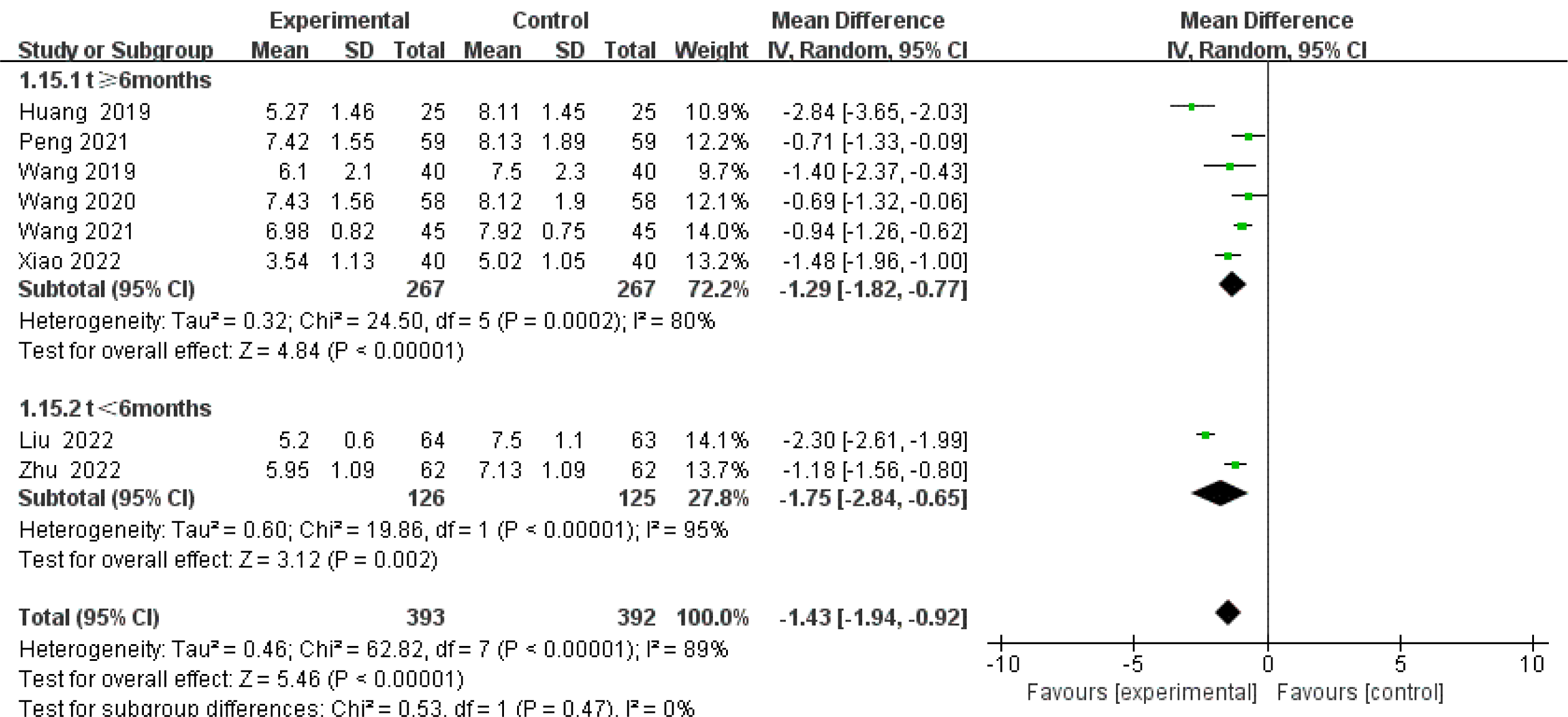

Supplement: Supplementary file 7 [file medi-102-e34958-s007.tif]

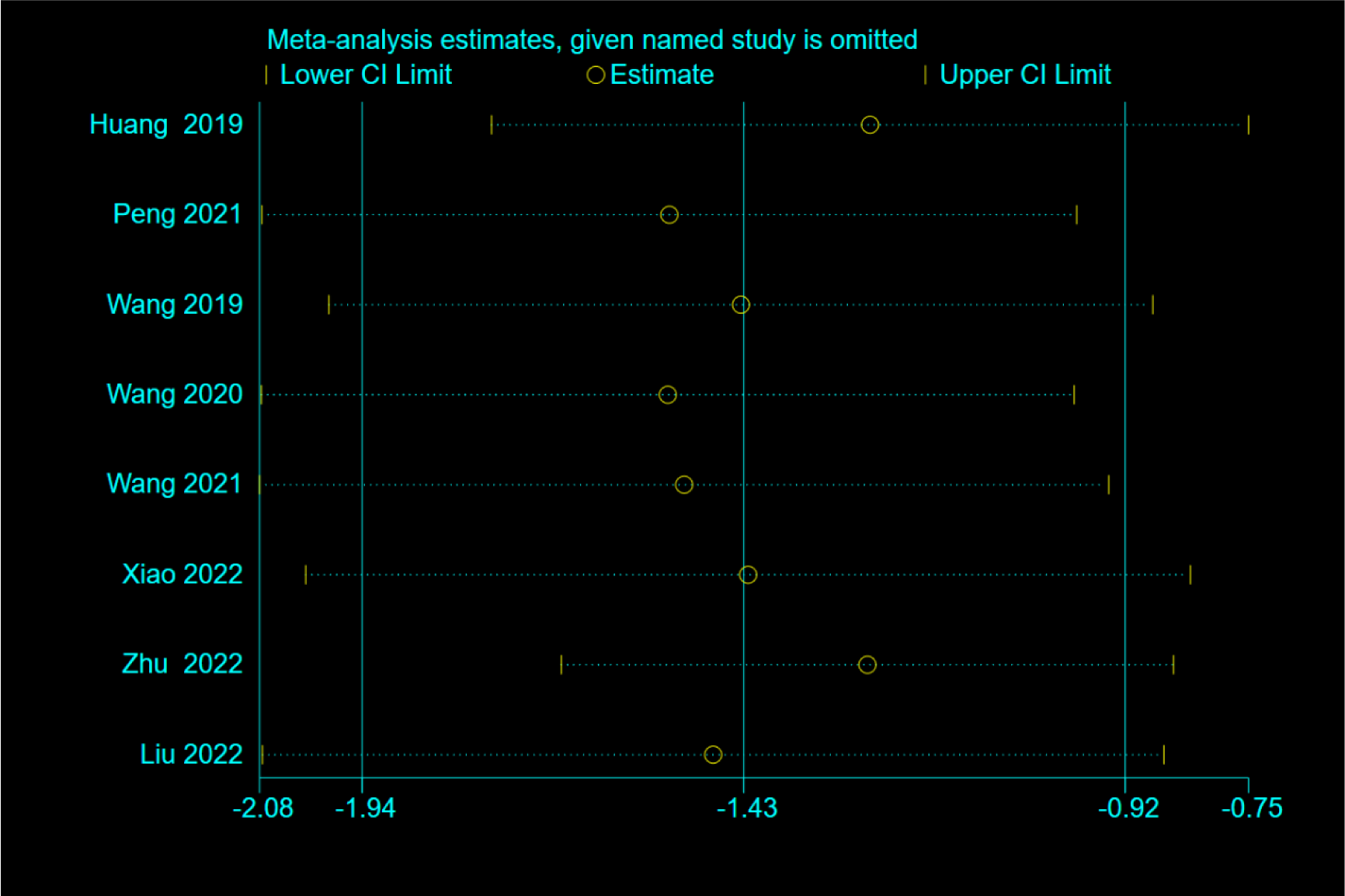

Supplement: Supplementary file 8 [file medi-102-e34958-s008.tif]

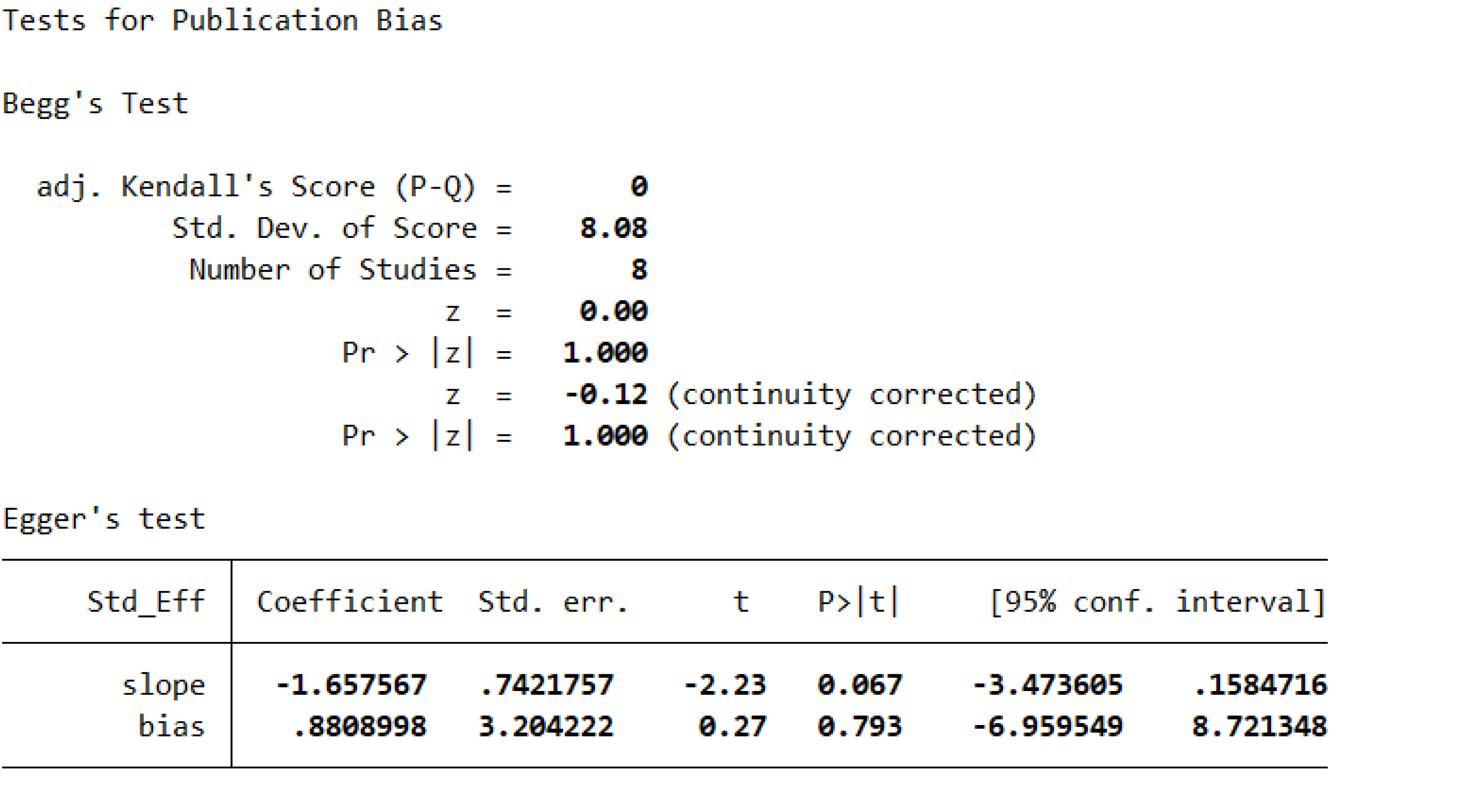

Supplement: Supplementary file 9 [file medi-102-e34958-s009.tif]

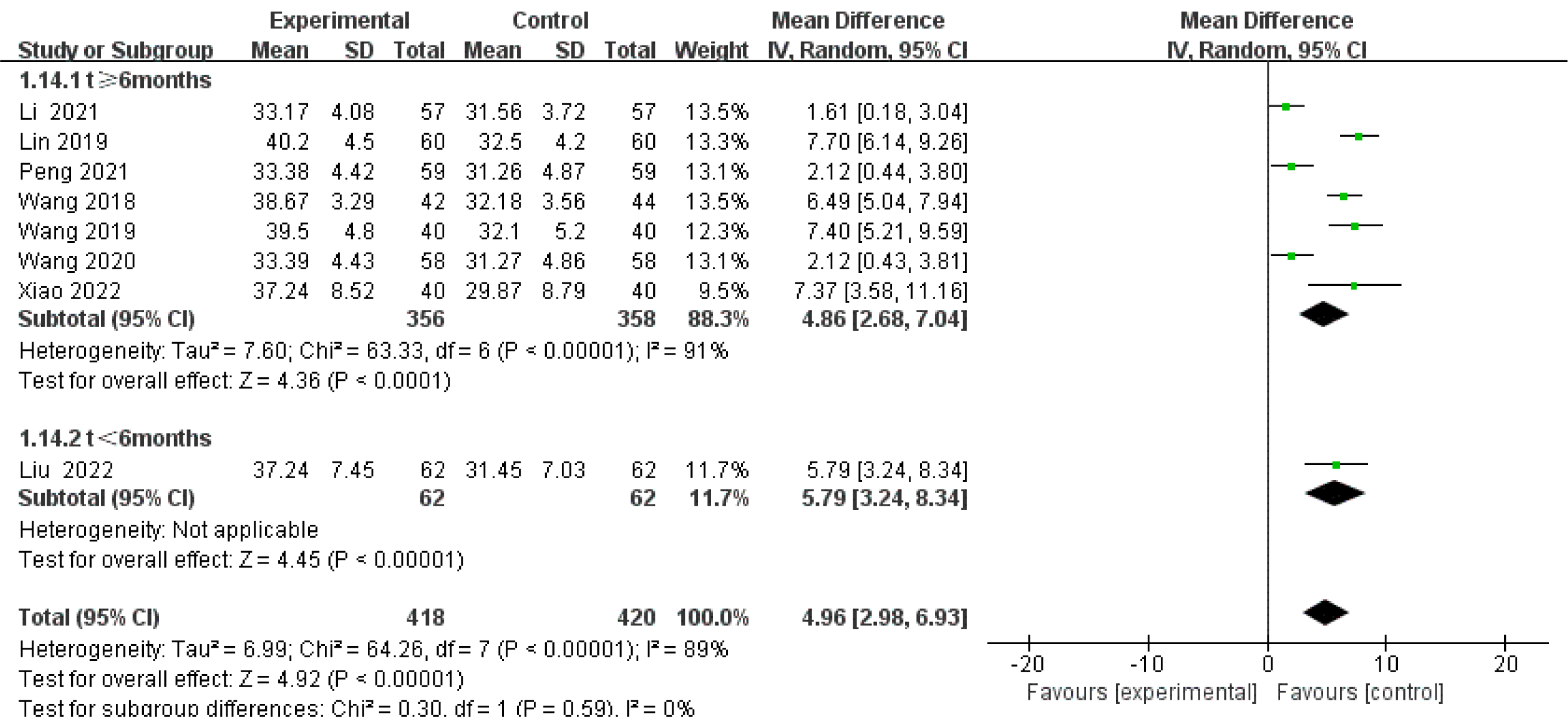

Supplement: Supplementary file 10 [file medi-102-e34958-s010.tif]

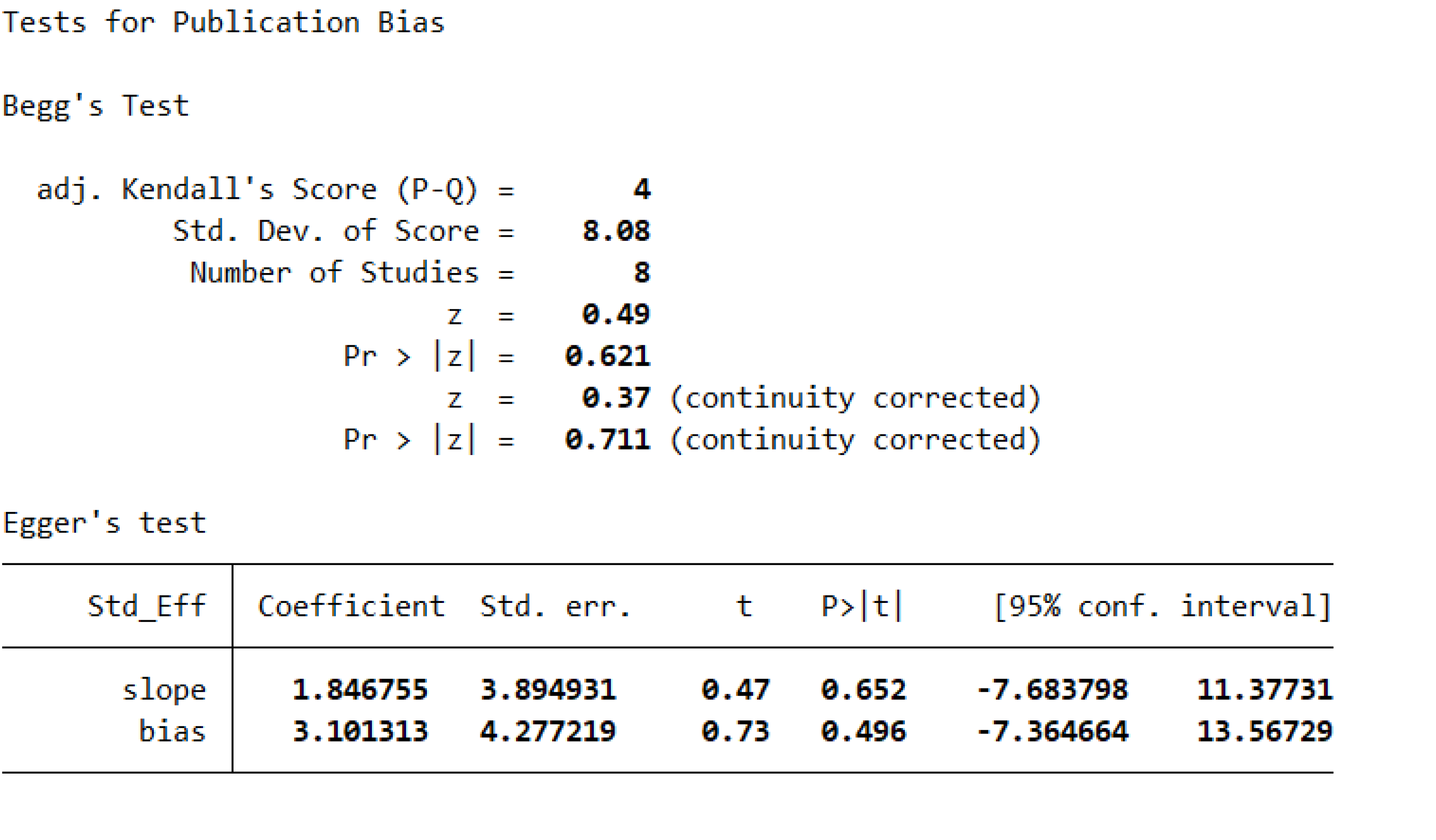

Supplement: Supplementary file 11 [file medi-102-e34958-s011.tif]

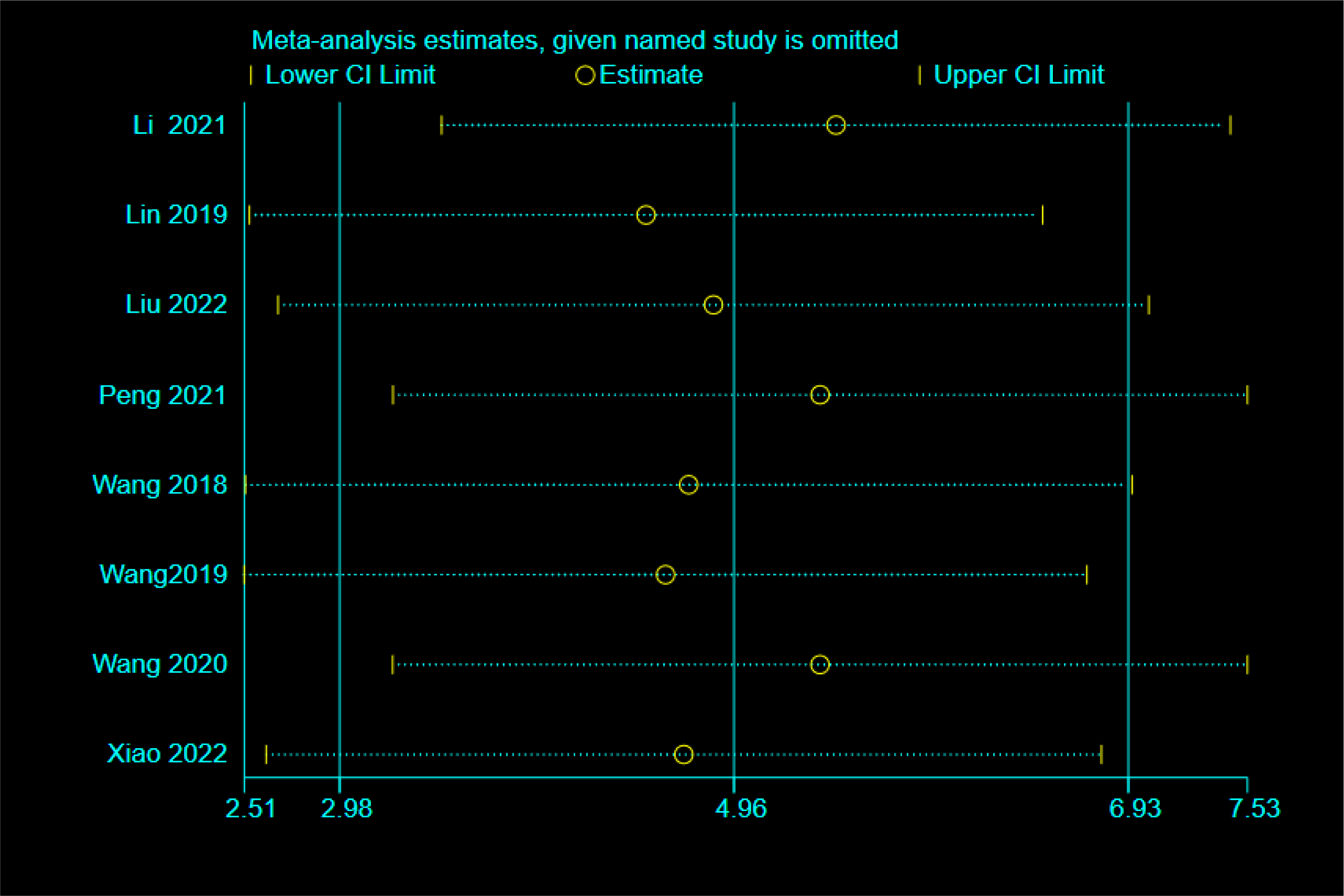

Supplement: Supplementary file 12 [file medi-102-e34958-s012.tif]

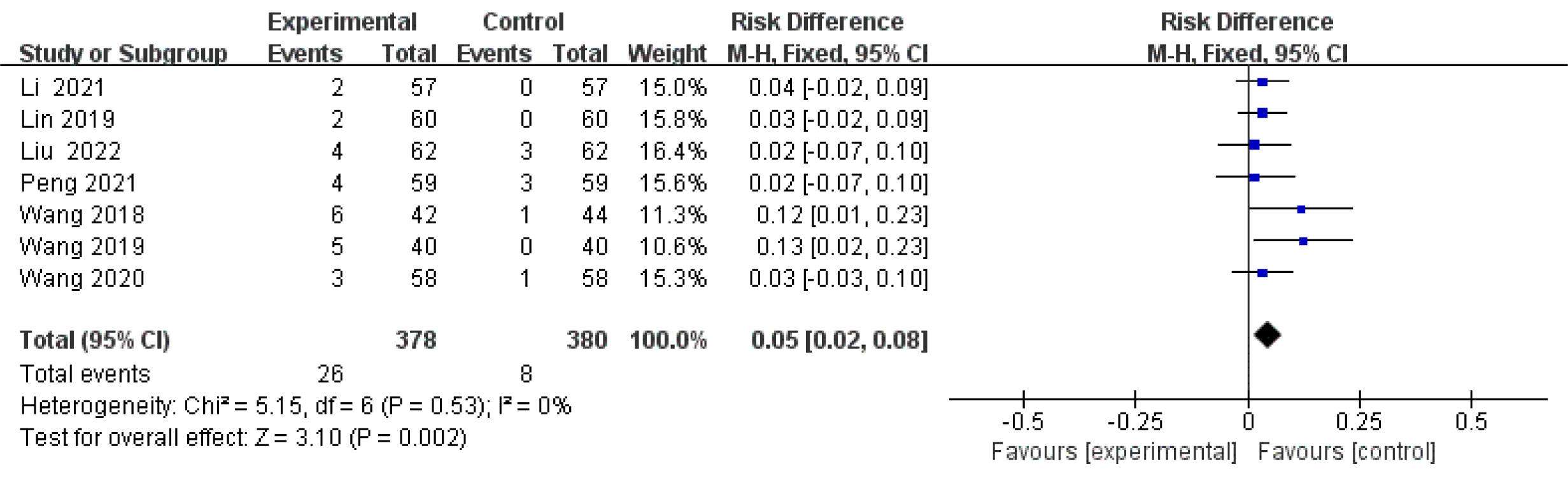

Supplement: Supplementary file 13 [file medi-102-e34958-s013.tif]
